# Annotations

**Published:** 1889-07-06

**Authors:** 


### ANNOTATIONS.

**Heat, Heads, Hats!**

*Negari non potest*, as the Latin primer says, or, as the average Briton would say, "unquestionably," the weather for some time has been awfully hot. Even the morning sun shining into the bedroom window has been powerful enough to waken the sleeper an hour before his time, whilst the morning walk to the station, for those who live in the country, at any rate, has necessitated the use of an umbrella for the protection of the head. Before June was out, the pastures were all baked dry; the corn in the fields was thin and pale from drought; the hops languished on their heated poles, and deaths from sunstroke were reported in the newspapers. About the weather in London and the neighbourhood, the general unanimity of opinion has been absolutely complete. A moderate degree of summer heat is not at all injurious to the general health, but, on the contrary, decidedly favourable. An immoderate and unseasonable fierceness of the sun's rays is, on the other hand, excessively exhausting, and to the head and nervous system is, in many cases, highly dangerous. It might be thought, therefore, that the average Briton, being, as he himself would say, a fairly sensible fellow, able to understand business, to show the world how to fight, to teach it how to colonise, setting an example of constitutional government, leading the way in the choice of a reasonable religion, avoiding extremes of all sorts; being, in short, the very quintessence of common sense, and the practical virtues—it might be thought that he would be equal to the present occasion and provide himself with headgear suited to the urgent necessities of the season. But *si monumentum quæris, circumspice*, which may be freely translated "If you wish to understand the natural pigheadedness of a Briton, look at his hat about Midsummer." The other morning the writer was crossing London Bridge at ten o'clock; the sun's rays were roasting, the river was poisonous, the dust was detestable. He looked at the hurrying crowds to see how they were dressed, and especially took note of their hats. Is it necessary to say that practically every man and boy had a black coat on, and that each and all, from the portly alderman with his rubicund nose to the bloodless and thin-legged clerk on fifty pounds a year, had on a hard black hat, and that in about ninety-nine per cent. of the cases that hat was of the "chimney-pot variety!" Vain was the attempt to discover a single white hat or a single soft one. Nothing but black, black, black, and hard, hard, hard, was anywhere to be seen. What is to be done? Reason appears to be out of the running; ridicule is absolutely powerless. John Bull, especially the Bull of the City, loves his shiny chimney-pot almost as dearly as his life. He thinks it is the mark and sign of his "gentility." Discomfort, danger, incongruity between the hat and the heat, what are they to him? Very well, John Bull; since you cannot, even to avoid the risk of death from sunstroke, dispense with your dearly-beloved black silk pot, stick to it by all means, and not only make yourself the laughing-stock of the world, but get your brains and your nervous system dangerously injured, and in some instances your life destroyed. Not even yourself can say it does not "serve you right."

**The Vaccination Commission Sitting in Private.**

AT the first sitting of the Royal Commission appointed to enquire into the Vaccination question, several representatives of the Press presented themselves, but were informed that the Commission would sit with closed doors. Inexperienced persons, and persons prone to be suspicious of their fellow-creatures, may think this augurs badly for the fairness of the enquiry that has been entered upon, and also for the value of the report which is looked forward to with so much interest by certain sections of the public. There can be no doubt, however, that in a case of this kind where so much warm feeling is displayed, at least by one party, it is better that the Commissioners should be freed from the distractions which a running fire of daily or weekly criticism would inevitably give rise to. The investigation is eminently one dealing with facts. The Commission must make sure that their report contains a solid basis of indisputable fact. Their recommendations will be of small consequence compared with the mass of fact they will surely bring together. It must be remembered that there are no very great difficulties in the way of the enquiry. The Vaccination Act has now been in operation a long time, quite long enough indeed for the development and accumulation of all kinds of experience, facts, and materials for comparison. Vaccination is no new thing introduced yesterday, and therefore necessarily of unproved value. Its age is numbered by scores of years, and the extent of its operation is almost worldwide. We can compare, for example, present with past generations of grown-up people and children. There are also available for comparisons populations which have been subjected to almost every degree of completeness in vaccination: countries, towns, and rural districts whose inhabitants have been efficiently vaccinated, inefficiently vaccinated, or not vaccinated at all. Of course, opponents of compulsory vaccination are at liberty to contend that though the Commission may demonstrate the value of vaccination as a preventive, that does not entitle it to recommend or Parliament to enact that the operation shall be everywhere compulsory by statute. But reasonable people will look at the question from the standpoint of plain sense. They will ask: Was smallpox a terrible and destructive scourge in this and all countries in times not at all remote? Is it now a disease that is seldom epidemic—never alarmingly so—and that, in fact, the average man who is efficiently vaccinated has little or no dread of? And has this terrible scourge gradually lost its terror-inspiring power as vaccination has become more general and more efficient? If it can be shown beyond reasonable doubt that such are the facts, the common sense of the majority will certainly say that if a proved pest and deadly scourge can be so easily and with so little risk kept in check by the compulsory vaccination of all children, all children for the sake of the community, and for the sake of themselves also, must be compulsorily vaccinated. We are for the most absolute freedom of speech, and for the utmost possible liberty of action compatible with the general well-being. But we cannot give our adhesion to a principle which would sacrifice the safety of the many to the unrestrained license of the few. While admitting that compulsory vaccination, in itself, is a proceeding which ought to excite the keenest criticism and the fullest enquiry, we cannot but feel that if criticism and enquiry vindicate the prevailing medical opinion of its value, Parliament, impelled by public opinion, will be bound to continue the Vaccination Act in force, and even to strengthen and extend it if it be necessary. Where people live together in communities the public safety is of necessity one of the primest considerations.

**The Horse's Turn.**

THE example of the Hospitals Association in originating a system of street ambulance for London has been contagious, and now we have Lady Frances Trevanion and Lady Florence Clinton stepping into the breach to save injured horses from the cruel and brutal fate which so often awaits them in the streets. Only those who daily traverse the crowded thoroughfares of the metropolis can have any idea how numerous, how severe, and how grievous are the accidents which happen to horses. Those docile friends of man are employed by tens of thousands in London for every conceivable purpose. From the most beautiful Arab to the sturdy drayhorse, and from the noble coachhorse to the humblest propeller of a four-wheeled cab, all are made available, and all have to run the gauntlet of the risks and dangers of the streets. The London horse knows by stern familiarity the meaning of the stress and strain of modern life. On him the problems of political economy work themselves out without hindrance and without remorse. The scientific law of the "survival of the fittest" is illustrated every day in his experience. A lover of horses cannot but heave a sigh of sadness as he thinks of the noble animals with so many masters and so few friends. It is much to be desired that Lady Frances Trevanion and Lady Florence Clinton will devote themselves earnestly and intelligently to the kindly and worthy duty they have taken in hand. They should place before the public at the earliest moment a year's facts of horse suffering in the metropolis. Such a statement, if it were anything like complete, would be a startling revelation to most people. Among the class to which these two ladies belong there is a large proportion of horse lovers. We urge upon them to give all possible sympathy and intelligent help to the new movement. A subscription list is already opened, and subscriptions may be sent to the honorary treasurer, Animals' Institute, 9, Kinnerton-street, Knightsbridge.

